# Clinical utility of anti-lipoarabinomannan antibodies testing for the diagnosis of tuberculous arthritis

**DOI:** 10.1186/s40064-015-0858-1

**Published:** 2015-02-05

**Authors:** Giuseppe Passiu, Gian Luca Erre, Pietro Pirina, Leonardo Antonio Sechi

**Affiliations:** Dipartimento di Medicina Clinica e Sperimentale, Università di Sassari, Sassari, Italy; Unità di Pneumologia, Università di Sassari, Sassari, Italy; Dipartimento di Scienze Biomediche, Università di Sassari, Sassari, Italy

**Keywords:** Arthritis, Mycobacteria, ELISA, Lipoarabinomannan

## Abstract

Diagnosis of extrapulmonary tuberculosis (TB) is often challenging. In this work we discuss the utility of an assay for Lipoarabinomannan (LAM) antibody detection in synovial fluid. LAM is one of the three major groups of lipopolysaccharides within the Mycobacterium tuberculosis (MTB) cell wall. An ELISA based test was used to investigate the presence of antibodies against LAM in an immunocompetent patient with knee arthritis. The symptoms resolved after isoniazid treatment. LAM positivity has been used as a diagnostic tool for TB in different settings, including veterinary field. The test could be of some value to diagnose tuberculous arthritis in selected patients when gold standard test returned negative although further investigations are welcome.

Tuberculosis (TB) remains one of the leading infectious cause of mortality world-wide. Musculoskeletal manifestations account for 10% of extrapulmonary tuberculosis and include tuberculous arthritis.

A 48-year-old otherwise healthy man presented with a 3-month long history of oligoarthritis (left hip and knees) preceded by three weeks of mild night sweats. Personal history was not informative, with the exception of a recent travel in a TB endemic area. General physical examination was unremarkable.

Intensive rheumatological work-up, including serology, complete joint imaging, chest X-rays and comprehensive synovial-fluid analysis was inconclusive. A diagnosis of undifferentiated arthritis was considered and two courses of intra articular steroid injection along with non steroidal antiinflammatory drugs were undertaken with only short relief.

Therefore, in the view of the recent history of TB contact and of IFN-gamma release assay positivity (19 U, cut off at 0.35 U) extensive search for *M. tuberculosis* on multiple biological specimens was performed: microscopy, polymerase chain reaction (PCR) and cultures of sputum, urine, synovial fluid and synovial tissue returned all negative. On the contrary, an intense positivity for anti-lipoarabinomannan (LAM) antibodies IgG (Erre et al. 2014) in synovial fluid, but not in peripheral blood was demonstrated.

A presumptive diagnosis of tuberculous arthritis was then considered and an expectancy therapy with a 6-month short course of isoniazide was started. Arthritis gradually improved in 6 months and after 10 months the end of treatment the patient is fully recovered.

TB diagnosis largely rely on clinical suspicion, smear staining, liquid culture for *M. tuberculosis* and nucleic acid amplification tests. The International Standards for TB Care strongly discourages the use of serological tests in routine practice for the diagnosis of pulmonary and extrapulmonary TB (Hopewell et al. 2006).

However, extrapulmonary TB may represent a diagnostic dilemma due to the relative insensitivity of smear, PCR and cultures of body fluid or tissues (Valdes et al. 1998). In TB arthritis synovial fluid smear staining is positive in only 20–40% of cases (Mahowald 2000) and 21% of the synovial fluid cultures have been reported to be negative (Malaviya & Kotwal 2003).

The accuracy of a commercial kit testing antibodies to LAM, a lipoglycan of the mycobacterial cell wall, has been recently tested showing a good discrimination power between TB and non-TB subjects (Malaviya & Kotwal 2003). The use of anti-LAM antibodies testing has been reported to be of utility in diagnosing pulmonary and extrapulmonary TB (Dhana et al. 2014; Yokoyama et al. 2005; Lawn et al. 2014; Bua et al. 2009).

In our case, the diagnosis of tuberculous arthritis could be established on the basis of patient history (including recent exposure to *M. tuberculosis* contacts), absence of an alternative clear diagnosis, positivity for anti-LAM antibodies on synovial fluid and complete recovery after anti-tuberculous chemotherapy.

Negativity of anti-LAM antibodies in peripheral blood, further strengthened the hypothesis of a true *M. tuberculosis* localization in the joint triggering anti-LAM antibodies production restricted to the synovial microenvironment.

The analysis of our case suggests that, anti-LAM testing could be of some value to diagnose tuberculous arthritis in selected patients when gold standard test returned negative. However, further investigations are required in the matter, before anti-LAM antibodies testing could be recommended as a routine TB diagnostic test.
